# Stomatin modulates the activity of the Anion Exchanger 1 (AE1, SLC4A1)

**DOI:** 10.1038/srep46170

**Published:** 2017-04-07

**Authors:** Sandrine Genetet, Alexandra Desrames, Youcef Chouali, Pierre Ripoche, Claude Lopez, Isabelle Mouro-Chanteloup

**Affiliations:** 1 Université Sorbonne Paris Cité, Université Paris Diderot, Inserm, INTS, Unité Biologie Intégrée du Globule Rouge, Laboratoire d’Excellence GR-Ex, 75739 Paris Cedex 15, France

## Abstract

Anion Exchanger 1 (AE1) and stomatin are integral proteins of the red blood cell (RBC) membrane. Erythroid and kidney AE1 play a major role in HCO_3_^−^ and Cl^−^ exchange. Stomatins down-regulate the activity of many channels and transporters. Biochemical studies suggested an interaction of erythroid AE1 with stomatin. Moreover, we previously reported normal AE1 expression level in stomatin-deficient RBCs. Here, the ability of stomatin to modulate AE1-dependent Cl^−^/HCO_3_^−^ exchange was evaluated using stopped-flow methods. In HEK293 cells expressing recombinant AE1 and stomatin, the permeabilities associated with AE1 activity were 30% higher in cells overexpressing stomatin, compared to cells with only endogenous stomatin expression. Ghosts from stomatin-deficient RBCs and controls were resealed in the presence of pH- or chloride-sensitive fluorescent probes and submitted to inward HCO_3_^−^ and outward Cl^−^ gradients. From alkalinization rate constants, we deduced a 47% decreased permeability to HCO_3_^−^ for stomatin-deficient patients. Similarly, kinetics of Cl^−^ efflux, followed by the probe dequenching, revealed a significant 42% decrease in patients. *In situ* Proximity Ligation Assays confirmed an interaction of AE1 with stomatin, in both HEK recombinant cells and RBCs. Here we show that stomatin modulates the transport activity of AE1 through a direct protein-protein interaction.

Stomatin, also known as protein 7.2 or as the major component of band 7, is a 31-kDa integral membrane protein expressed at the membrane of red blood cells (RBCs)^1^,^2^. This protein was named according to the rare human haemolytic anaemia hereditary stomatocytosis^3^. The corresponding gene (*EPB72)* encodes a member of highly conserved and ubiquitously expressed proteins. Models of the predicted protein structure showed a short cytosolic N-terminal head, a strongly hydrophobic 28-amino acid stretch presumably encoding an amphipathic helix embedded in the cytosolic leaflet of the bilayer and a large cytosolic C-terminal domain composed of beta sheets and alpha helices. A part of this last domain is common to stomatin-like and related proteins and is referred as SPFH (stomatin, prohibitin, flotillin, HflC/K)-domain^4^,^5^ or PHB (prohibitin homology)-domain^6^. Crystal structures of a SFPH-domain of mouse stomatin revealed typical banana-shaped dimers which can further assemble via two conserved surfaces into a cylindrical oligomer^7^. Stomatin interacts with various ion channels and modulates their activities^7^,^8^,^9^. As regards the acid-sensing ion channel 3 (ASIC3), an accurate relationship was described between the inhibition of the channel and the oligomeric state of the SFPH-domain of stomatin. In RBCs, using mass spectrometry of the isolated cross-linked stomatin complexes, potential interaction partners of stomatin were detected^10^. The identification, as stomatin partners, of the glucose transporter (GLUT1), as well as of anion exchanger 1 (AE1) and water channel aquaporin-1 (AQP1) suggests that stomatin within cholesterol-rich membrane domains plays a role as a membrane-bound scaffolding protein modulating transport proteins. While an interaction with GLUT1 is consistent with the previously demonstrated role of stomatin in regulating the switch from glucose to L-dehydroascorbic acid (DHA) transport in humans and a few other mammals^11^, our previous study performed on RBCs that express stomatin or not showed that the AQP1-mediated water transport across the membrane was unmodified in stomatin-deficient erythrocytes^12^. Concerning AE1 activity, to date, no study had demonstrated a role of stomatin in the chloride/bicarbonate exchange.

AE1, the anion exchanger 1, also known as band 3 or solute carrier family 4 member A1 (SLC4A1), is a transmembrane protein that facilitates Cl^−^/HCO_3_^−^ exchange across the plasma membrane of both RBCs and alpha intercalated cells in the kidney (reviewed in ref. 13). Consequently, it participates in the regulation of the intracellular pH, ion and volume homeostasis of the erythrocyte^14^ and in acid secretion in the kidney. In RBCs, this 95-kDa glycoprotein also exhibits a critical role in the membrane structure due to its presence in two major multiprotein complexes. One complex is composed of a dimer of band 3 (junctional membrane complex including actin) while the second contains a tetramer of band 3 (ankyrin complex), both of them being anchored to the spectrin-based skeleton^15^,^16^. While 20–25% and 40% of total AE1 proteins are located within the junctional and the ankyrin complexes, respectively, the remaining AE1 (25–30%) is present as free dimers in the RBC membrane^17^. Several hereditary hemolytic anemia (spherocytosis^18^ or stomatocytosis^19^) are a consequence of mutations in AE1 or in members of these complexes, leading to anchorage abnormalities and erythrocyte diseases. The AE1 protein is composed of two main domains: a cytosolic N-terminal domain (cdb3) (1–359 amino acids) and a membrane C-terminal domain (mdb3) (360–911 amino acids). The cdb3 domain for which a crystal structure was previously described^20^ possesses binding sites for ankyrin R^21^, protein 4.2^22^, adducin^23^ and protein 4.1^24^ in addition to glycolytic enzymes^25^,^26^ and haemoglobin^27^. Kidney AE1 (kAE1) exhibits a truncation of the 65 first N-terminal amino acids in the cdb3 which removes the binding site to ankyrin R^28^. As regards the mdb3 which corresponds to the physical site of the chloride/bicarbonate exchange, the recent crystal structure of this functional domain^29^ allowed the identification of the anion-binding position in AE1 and showed the proximity of natural mutations that lead to diseases^30^.

We recently developed a stopped-flow based-assay in order to investigate the bicarbonate transport across the RBC membrane or plasma membrane of eukaryotic cells expressing recombinant AE1^31^. AE1 transport activity was tested in that paper using ghosts derived from the RBCs of a patient presenting a hereditary stomatocytosis (HSt) with a normal expression of stomatin. This pathology is associated with the G796R mutation in AE1^32^ and, in accordance with the heterozygous status of the patient, RBC exhibited a 50% reduction of the AE1 activity using our functional assay. Moreover, this stopped-flow approach was used in the same study to assess the AE1 activity on HEK293 cells expressing the recombinant protein, using an inducible expression system which enables obtaining controlled and stable expression levels of AE1 at the membrane^31^.

Furthermore, the availability of rare stomatin-deficient RBC samples from several patients presenting RBC membrane disorders and corresponding to previously described mutations in either RhAssociated Glycoprotein (RhAG) (F65S) for overhydrated stomatocytosis (OHSt)^33^ or GLUT1 (G286D) for a cryohydrocytosis (CHC)^34^, presented a unique opportunity to investigate the influence of stomatin in the AE1 activity.

In the present study, chloride/bicarbonate exchange mediated by AE1 was examined in both stomatin-deficient RBCs and HEK293 cells expressing recombinant AE1 and overexpressing stomatin, by measurement of rapid fluxes of bicarbonate and chloride. Furthermore, the interaction between AE1 and stomatin, in both RBCs and HEK293 cells was demonstrated by proximity ligation assays (PLA).

## Results

### Decrease of the AE1 activity in stomatin-deficient RBCs

**Figure 1:**
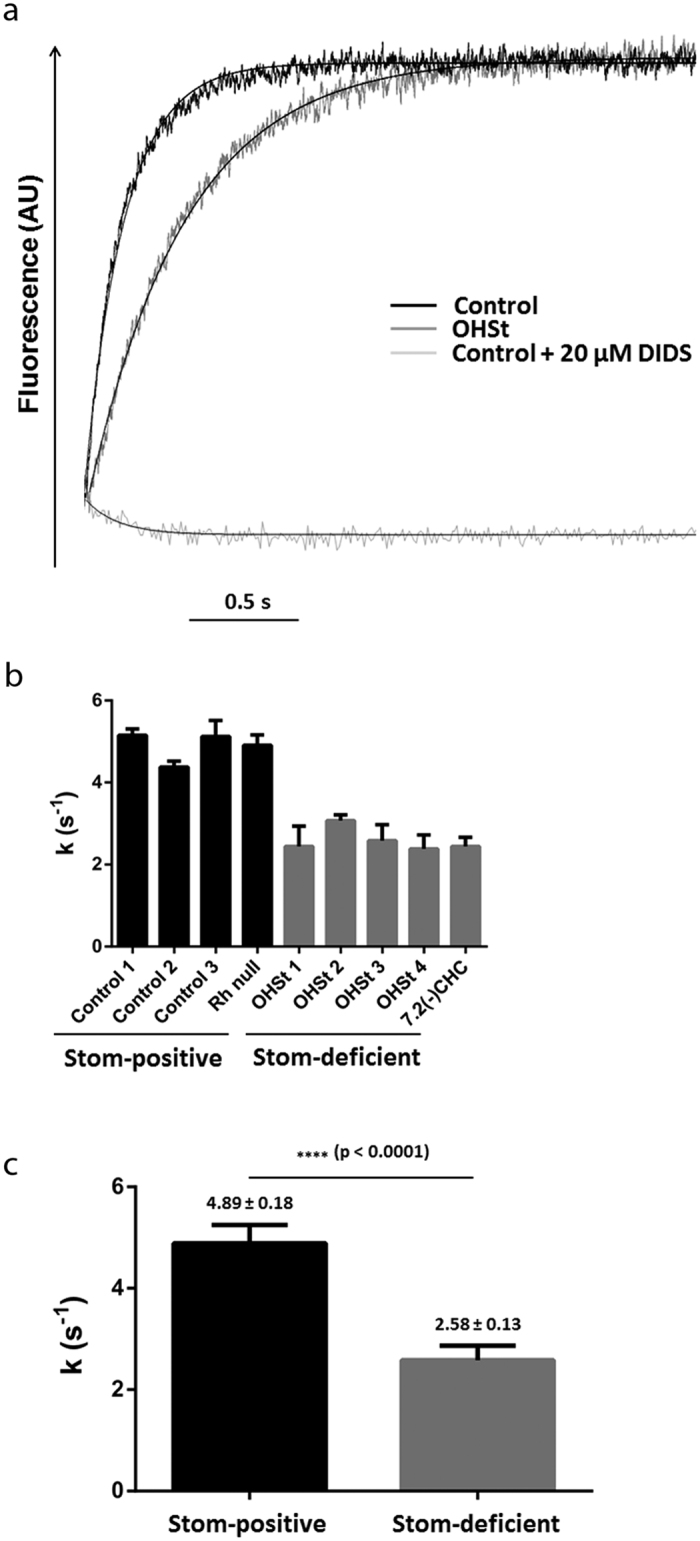
Cl^−^/HCO_3_^−^ exchange activity across control or stomatin-deficient RBC membranes estimated from pHi changes. (**a**) Individual time-courses of fluorescence and pHi changes in ghosts prepared from RBCs of a control and an OHSt patient. Ghosts resealed in the presence of a pH-sensitive dye (pyranine) were submitted to 100 mEq outwardly-directed chloride and 50 mEq inwardly-directed bicarbonate gradients at 30 °C in the stopped-flow spectrofluorometer. As a specificity control, ghosts were incubated with 20 μM DIDS for 30 minutes prior to analysis. Data from three to four time-courses were averaged. Smooth lines are monoexponential fits to the data using the simplex procedure of the Biokine (Bio-Logic), providing alkalinization rate constants (k, s^−1^). (**b**) At least three experiments for each patient (Stom-positive: four controls and one Rhnull (black bars), Stom-deficient: four OHSt and one 7.2(−)CHC patient (grey bars)) were averaged and can be compared. (**c**) Means of the alkalinization rate constants (k, s^−1^) ± SEM for Stom-positive and Stom-deficient were reported. p value was determined using an unpaired Student t test.

In order to investigate the impact of stomatin deficiency in RBCs on the activity of AE1, a stopped-flow assay based, as previously described^31^, on the measurements of the kinetics of pHi changes related to HCO_3_^−^ entry was applied to ghosts derived from four OHSt and one 7.2(−)CHC patients. Since the pHi measurement, using a fluorescent probe, is very sensitive, inconstant residual endogenous carbonic anhydrase resulting from the ghost preparation could non-homogeneously enhance the chloride/bicarbonate exchange. However, after the addition of 2 mg/ml of bovine carbonic anhydrase, HCO_3_^−^ entry could be then accurately compared between several ghost samples (discussed in ref. 31). As shown in Fig. 1a from the individual time-courses of fluorescence changes in the ghosts of one control and one OHSt (black and grey curves, respectively), an alkalinisation was obtained for both samples. However, the kinetics was slower in ghosts derived from the OHSt patient. The alkalinisation rate constants k were deduced for each patient (Stom-positive: four controls and one Rhnull; Stom-deficient: four OHSt and one 7.2(−)CHC patient) and compared (Fig. 1b), showing a clear decrease of these constants k in Stom-deficient, when compared to Stom-positive samples. A significant decrease of 47% could be thus calculated from the means of these constants (Fig. 1c, Stom-deficient: 2.58 ± 0.13 s^−1^ and Stom-positive: 4.89 ± 0.18 s^−1^). This decrease can be strictly correlated to a diminution of the AE1 activity, since similar expression levels of AE1 were previously demonstrated in Stom-deficient and Stom-positive RBCs^12^.

**Figure 2:**
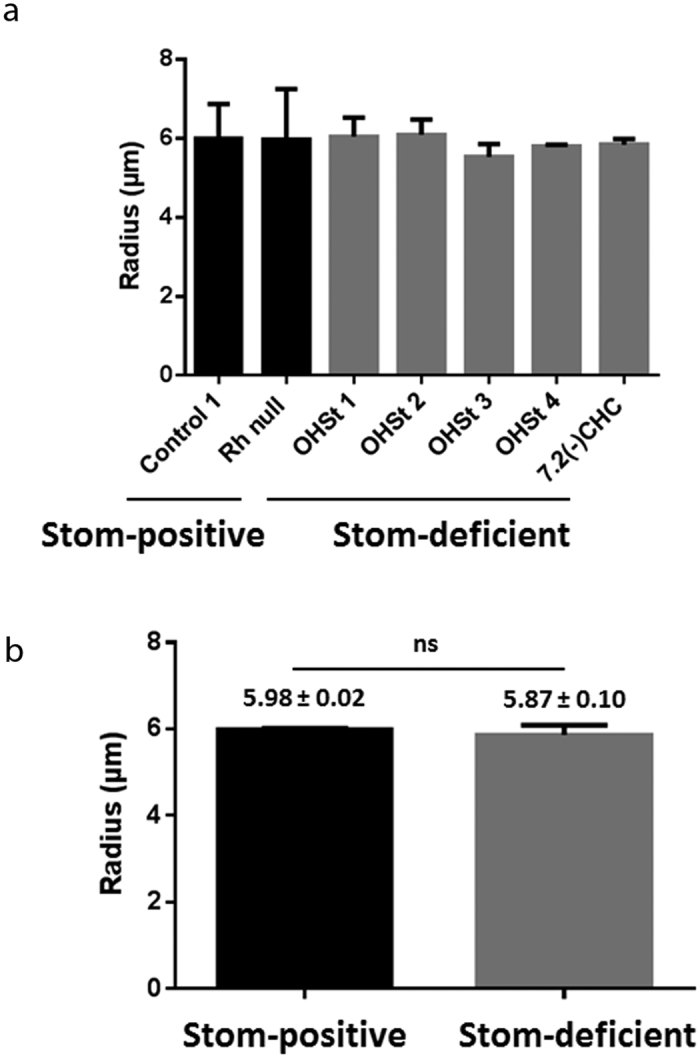
Analysis of the size of ghosts. (**a**) Means of ghost radius from two stomatin-positives (black bars), including one control and one Rhnull and five stomatin-deficients (grey bars), including four OHSt and one 7.2(−) CHC (+/− SEM). Measurements of 300 ghosts per individual from Zeiss Axio Observer Z1 microscope observations (X1000). (**b**) The comparison of stomatin-positive (black bars) and stomatin-deficient (grey bars) and determination of the means of the radius (μm) ± SEM revealed no significant (ns) differences corresponding to p > 0.05 (0.5082), as determined with an unpaired Student t test.

To compare the apparent permeability to HCO_3_^−^ (P’_HCO3-_) across the RBC membranes of different samples, not only the constant k but also V and SA, the volume and the surface area of the ghosts respectively, should be determined. Because the apparent permeability must satisfy the equation P’_HCO3−_ = k.V/SA and because, ghosts being considered as spheres, this equation could be simplified into P’_HCO3−_ = k.r/3, the radius r was estimated for each ghost sample (Fig. 2a). Interestingly, the radius values were similar for all samples (Fig. 2b), although the cell volume in OHSt patients is higher than that of controls. Consequently, the permeability to HCO_3_^−^ is directly proportional to the constant k, allowing the deduction of a 47% reduction of this permeability in Stom-deficient, when compared to Stom-positive samples.

**Figure 3:**
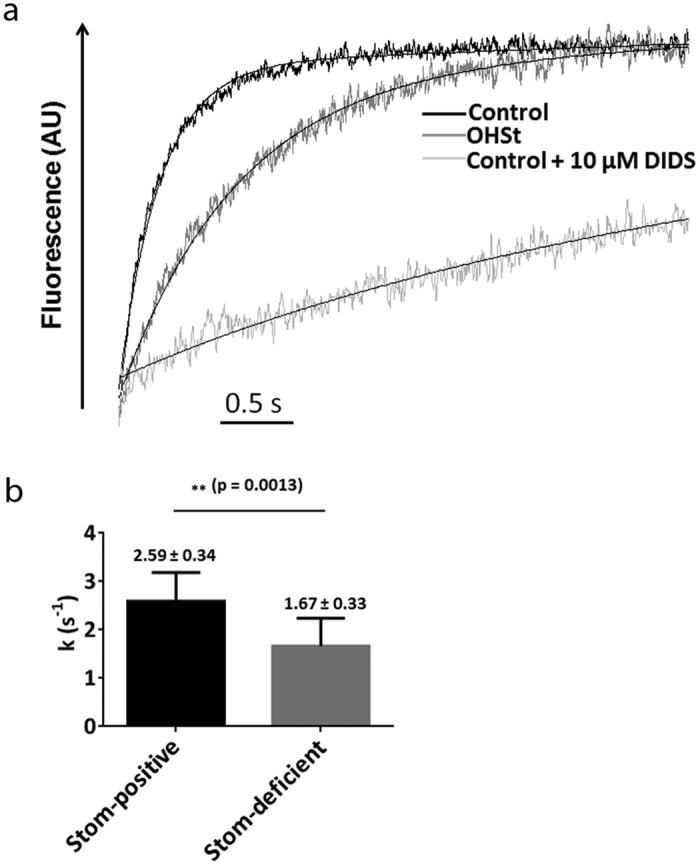
Cl^−^/HCO_3_^−^ exchange activity across control or stomatin-deficient RBC membranes estimated from intracellular chloride concentration changes. (**a**) Individual time courses of fluorescence and probe dequenching by the decrease of the chloride concentration in ghosts prepared from control and stomatin-deficient RBCs. Ghosts resealed in the presence of a chloride-sensitive dye (SPQ) were submitted to 100 mEq outwardly directed chloride and 25 mEq inwardly directed bicarbonate gradients at 20 °C in the stopped-flow spectrofluorometer. As a specificity control, ghosts were incubated with 10 μM DIDS for 30 minutes and centrifuged in order to remove unbound DIDS prior to analysis. Data from three to four time-courses were averaged. Smooth lines are monoexponential fits to the data using the simplex procedure of the Biokine (Bio-Logic), providing probe dequenching rate constants (k, s^−1^). (**b**) Three independent experiments allowed the determination of an average of the rate constants k for four controls (Stom-positive) and three OHSt (Stom-deficient). From these averages, means ± SEM were calculated for Stom-positive (black bars) and Stom-deficient (grey bars) and were compared using a paired Student t test (p = 0.0013).

In the present study, not only HCO_3_^−^ entry was analysed but also chloride exit. For this purpose, a new stopped-flow based approach was developed, using a chloride-sensitive dye (SPQ) and adapted from a method previously described^35^. Activation energy was determined (Fig. 1S), giving a value (Ea = 26.64 kcal/mol) similar to that previously reported by studies performed with ^36^Cl (20 kcal/mol) 36 and consistent with the activity of an exchanger. In these chloride transport experiments, no carbonic anhydrase was added. However, because of a potential enhancing effect of residual endogenous carbonic anhydrase in ghosts, as discussed above, acetazolamide which is an inhibitor of carbonic anhydrase was initially added to the ghosts before the stopped-flow experiments. As no modification of the kinetics was observed in the presence of acetazolamide either in controls or patients (data not shown), the addition of acetazolamide was considered dispensable in the following experiments. As shown in Fig. 3a, from the individual time-courses of fluorescence changes in the ghosts of one control and one OHSt, a probe dequenching by the decrease of the chloride concentration was obtained for both samples. However, the kinetics was once again slower in ghosts derived from the OHSt patient. The probe dequenching rate constants k were deduced in three independent experiments and averaged for three OHSt (Stom-deficient) and three controls (Stom-positive). From the means of these averages (Stom-deficient: 1.67 ± 0.33 s^−1^ and Stom-positive: 2.59 ± 0.34 s^−1^), a significant decrease of 42% could be calculated (Fig. 3b). Furthermore, Ea were calculated for the chloride permeation in ghosts derived from the RBCs of a control (Ea = 26.64 kCal/mol) (Fig. 1S) and of an OHSt (Ea = 23.54 kcal/mol)(data not shown), giving equivalent values.

### Interaction of AE1 and stomatin in control RBCs

**Figure 4:**
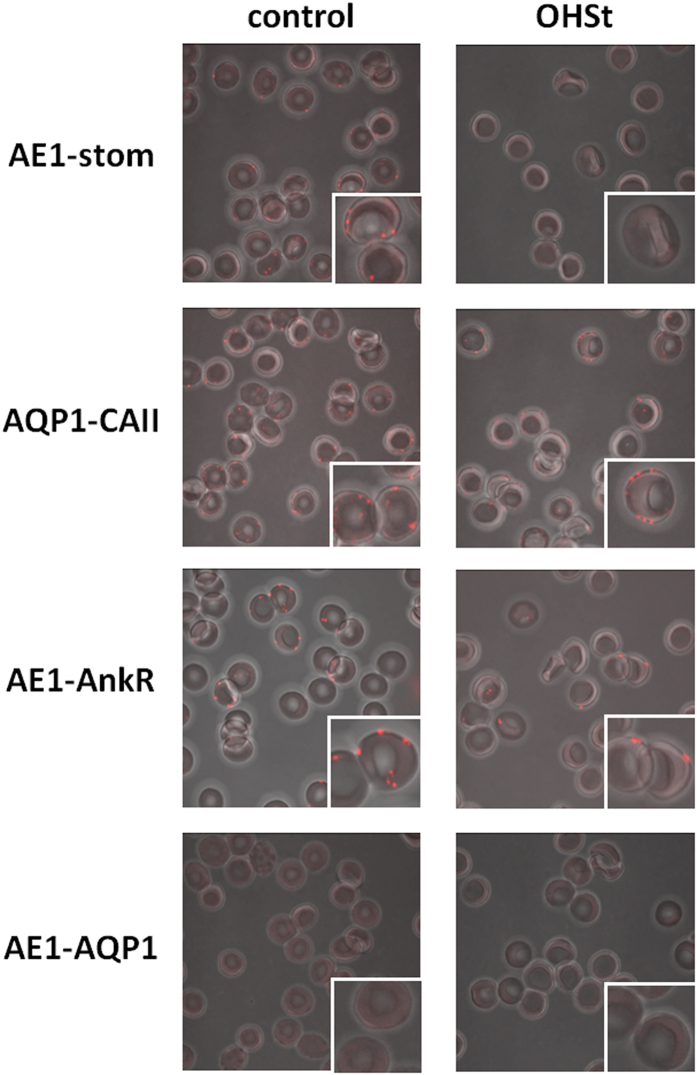
Proximity ligation assays for protein interaction between AE1 and stomatin (AE1-stom). RBCs from a control (stomatin-positive) and an OHSt (stomatin-deficient) patient were treated as described in experimental procedures, using anti-AE1 along with anti-stomatin antibodies and anti-AE1 along with anti-ankyrin R antibodies. For controls, two other pairs of antibodies were used: anti-AQP1 along with anti-CAII (positive controls) and anti-AE1 along with anti-AQP1 (negative controls). After PLA, RBCs were examined by confocal microscopy using a Zeiss LSM700 inverted confocal microscope equipped with a x100 oil-immersion objective, numerical aperture 1.4. Z-stack confocal image capture was performed and analyzed using the ZEN software.

Stomatin-positive (control) and stomatin-deficient (OHSt) RBCs were subjected to a proximity ligation assay (PLA) for protein interaction between AE1 and stomatin, as described in experimental procedures. The pictures (Fig. 4) show the merged images of a bright-field viewing of RBCs from a control and an OHSt to a maximum intensity projection of PLA signals visualized as red spots. For the AE1-stomatin PLA, a signal was clearly visible in stomatin-positive (control) and absent in stomatin-deficient (OHSt) RBCs. This result confirms interactions between stomatin and AE1 in control RBCs, the absence of stomatin in OHSt consequently resulting in absence of PLA signal in the patient RBCs. It is noteworthy that the number of red spots does not quantitatively match with the expected number of interaction sites and is much lower than that obtained in PLA on HEK293 cells (see below). Several PLA controls were also carried out. The association of anti-AQP1 with anti-CAII gave, as expected, a positive PLA in the RBCs of both the control and the OHSt. Indeed, these results confirm, for the first time in RBCs, the observations of Vilas *et al*.^37^, that clearly showed by PLA that recombinant AQP1 and endogenous CAII were interacting in HEK293 cells. Anti-AE1 used along with anti-AnkR resulted in a positive PLA in control erythrocytes as well as in OHSt. In contrast, when anti-AE1, which gave a PLA signal with anti-stomatin or anti-AnkR, was used with anti-AQP1, which gave a PLA signal with anti-CAII, a negative PLA response was obtained in both control and OHSt RBCs. This indicates a distance >40 nm between AE1 and AQP1 and is consistent with the absence of AQP1 from the multimolecular AE1 complex.

### Increase of the AE1 activity in HEK293 cells overexpressing stomatin

**Figure 5:**
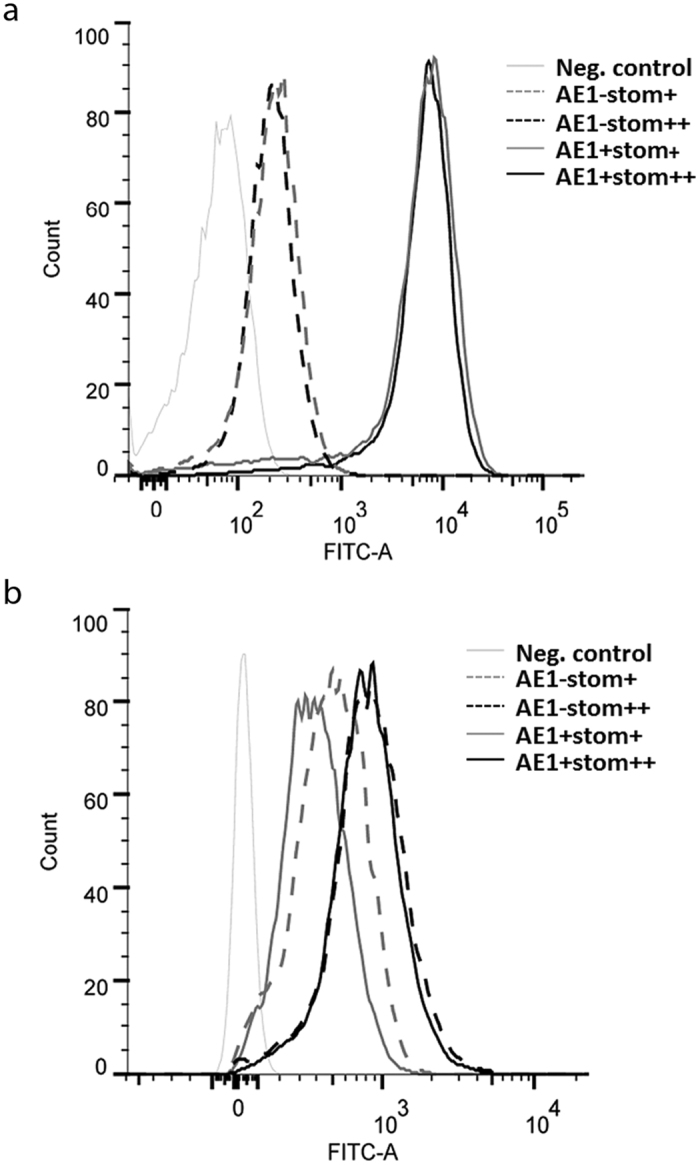
Expression of recombinant AE1 (**a**) and stomatin (**b**) in transfected HEK 293 cells. Recombinant AE1 was detected at the surface of the non-permeabilized cells by flow cytometry using mouse monoclonal BRIC6 antibody directed against AE1 extracellular domains. After cell fixation and permeabilisation, recombinant and/or endogenous stomatin was detected using goat polyclonal M-14 antibody. AE1− and AE1+ are non-induced or induced cells for AE1 expression, respectively. Stom + and stom ++ correspond to cells expressing endogenous and endogenous plus recombinant stomatin, respectively.

In order to test the effect of overexpression of stomatin on the AE1 activity, recombinant HEK293-AE1 cells were transfected with plasmid pCEP4-stomatin. The transfection and tetracycline induction system (described in Experimental Procedures) gave rise to four types of cells: endogenous stomatin-expressing cells, with or without tetracycline-induction of AE1 (AE1+ Stom+ or AE1-Stom+), and recombinant stomatin-expressing cells, with or without tetracycline-induction of AE1 (AE1+ Stom++ or AE1-Stom++). As shown by flow cytometry experiments using anti-AE1 antibody (BRIC6) directed against an extracellular epitope of the protein, AE1 was, as expected, only expressed after tetracycline-induction (Fig. 5a, AE1+ Stom+ and AE1+ Stom++). More interestingly, this expression was detected at a similar level whether or not the cells were transfected with pCEP4-stomatin. Flow cytometry experiments using anti-stomatin antibody also revealed the endogenous expression of stomatin in HEK293 cells whether or not these cells were induced by tetracycline (Fig. 5b, AE1+ Stom+ and AE1-Stom+). After transfection with pCEP4-stomatin, a similar overexpression of stomatin was obtained whether or not the cells expressed recombinant AE1. Indeed, in these two types of cells (AE1+ Stom++ and AE1-Stom++), the expression level of stomatin exhibited a two-fold increase when compared to that of cells only expressing the endogenous stomatin (AE1+ Stom+ and AE1-Stom+).

**Figure 6:**
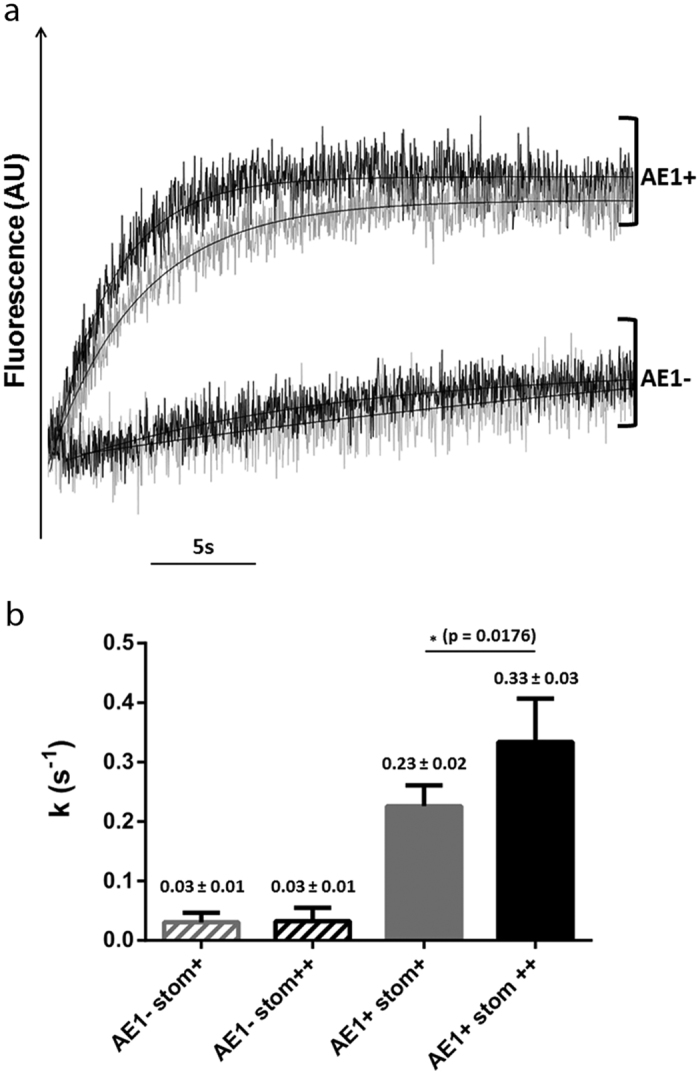
Cl^−^/HCO_3_^−^ exchange activity in HEK293 cells expressing inducible AE1 protein. (**a**) Time-course of fluorescence and pHi changes was monitored in HEK293 cells expressing (AE1+) or not AE1 (AE1−), with endogenous (grey) or endogenous plus recombinant (black) stomatin. Cells loaded with the fluorescent pH-sensitive probe BCECF-AM were submitted to inwardly-directed 10 mEq HCO3^−^/CO_2_ and outwardly-directed 67.5 mEq Cl^−^ gradients at 30 °C. Data from five to eight time-courses were averaged. Smooth lines are monoexponential fits to the data using the simplex procedure of the Biokine (Bio-Logic), providing alkalinization rate constants (k, s^−1^). (**b**) Values are means of constant k obtained in five independent experiments ± SEM. p values were determined with an unpaired t test from means obtained with induced cells. AE1− and AE1+ are non-induced or induced cells for AE1 expression, respectively. Stom+ and stom++ correspond to cells expressing endogenous and endogenous plus recombinant stomatin, respectively.

The Cl^−^/HCO_3_^−^ exchange activity of AE1 was measured and analysed in the four types of cells (AE1+ Stom++, AE1+ Stom+, AE1-Stom++, AE1-Stom+), using the stopped-flow approach, as previously described^31^. As expected, time-courses of fluorescence (Fig. 6a) clearly showed a rapid alkalinisation only when cells were expressing AE1 after tetracycline-induction (AE1+). However, the kinetics of pHi changes corresponding to an increase of the fluorescence intensity was more rapid in cells overexpressing stomatin (AE1+ Stom++, black curve). As described in experimental procedures, the alkalinisation rate constants k could be deduced from the exponentials that fit to the experimental curves. Five independent experiments yielded means of alkalinisation rate constants (k, s^−1^). While a similar background of 0.03 ± 0.01 s^−1^ was obtained in both AE1-Stom+ and AE1-Stom++, a significant increase of the AE1 activity (30.3%) was revealed in cells overexpressing stomatin (AE1+ Stom+: 0.23 ± 0.02 s^−1^ and AE1+ Stom++: 0.33 ± 0.03 s^−1^), indicating an impact of the expression level of stomatin on the Cl^−^/HCO_3_^−^ exchange mediated by AE1 (Fig. 6b).

### Interaction of AE1 and stomatin in HEK293 cells

**Figure 7:**
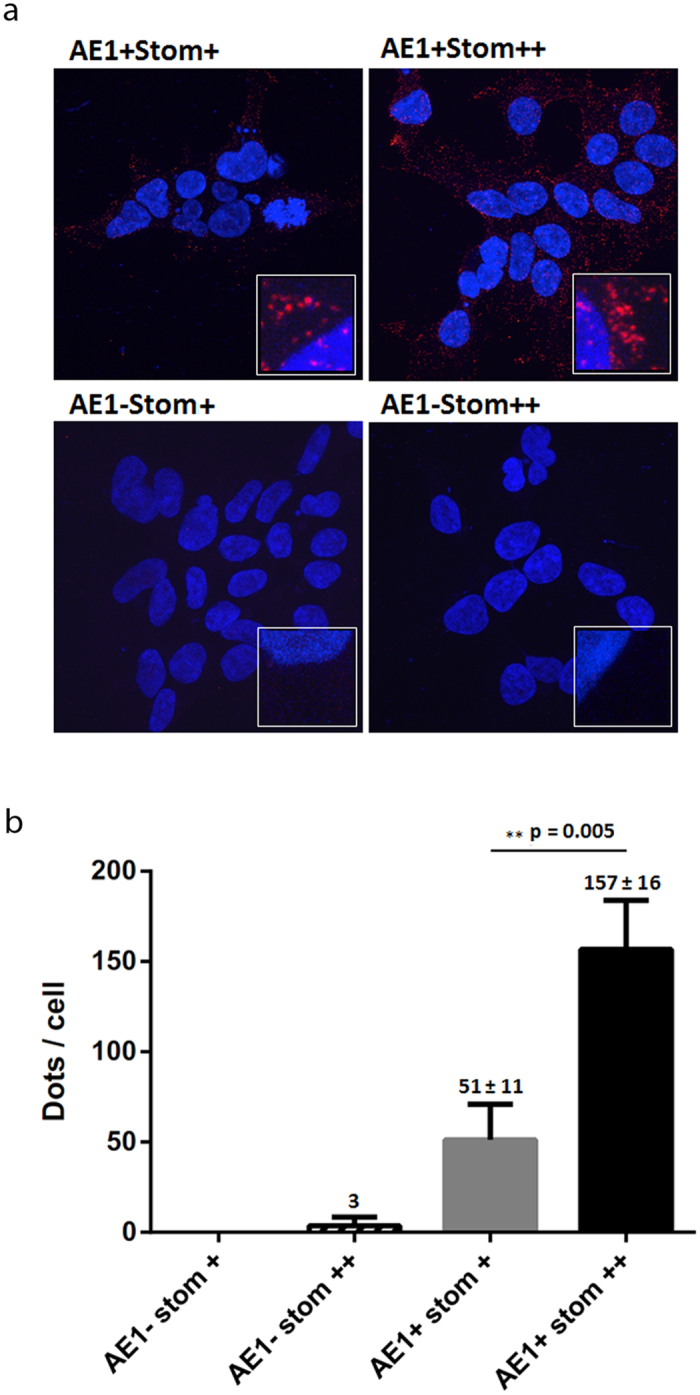
Proximity ligation assay for protein interaction between AE1 and stomatin in HEK293 cells expressing inducible AE1 protein (**a**) and dots/cell quantification (**b**). Cells previously grown on poly-L-lysine coverslips were either tetracycline-induced (AE1+) or non-induced (AE1−), with endogenous (Stom+) or endogenous plus recombinant (Stom++) stomatin. Cells were probed with rabbit anti-AE1 and goat anti-stomatin, and then subjected to PLA. Samples were mounted in ProLong Gold antifade reagent with DAPI, allowing cell nuclei staining, and examined by confocal microscopy using a Zeiss LSM700 inverted confocal microscope equipped with a x100 oil-immersion objective, numerical aperture 1.4. Z-projected images of the collected z-stack were performed and analyzed using the ZEN software. Inset: magnification (x16) of a selected region of PLA image. Each red spot represents for a single interaction. Experiments were repeated three times for AE1+ Stom+ and AE1+Stom++ to obtain the number of dots per cell, with a total of 35 and 40 individual cells counted, respectively. Values are means ± SEM. p value was determined with an unpaired Student t test.

The four types of cells (AE1+ Stom++, AE1+Stom+, AE1-Stom++, AE1-Stom+) were subjected to a proximity ligation assay (PLA) for protein interaction between AE1 and stomatin. The pictures (Fig. 7a) show maximum intensity projection of the raw confocal images based on collected z-stack images. PLA signals are shown in red and the nuclei in blue, allowing a quantification of dots/cell. As expected, non-induced cells (AE1-Stom+, AE1-Stom++) were totally devoid of red spot, since AE1 was not expressed. In contrast red spots were visualized in tetracycline-induced cells (AE1+Stom+, AE1+Stom++), showing an interaction between recombinant AE1 and stomatin. Interestingly, the number of spots was significantly higher, as shown in Fig. 7a and after quantification by ImageJ software on three independent experiments (Fig. 7b), in cells overexpressing (endogenous plus recombinant) stomatin (AE1+ Stom++) compared to cells expressing only endogenous stomatin (AE1+ Stom+).

## Discussion

The present findings provide a new insight on the participation of stomatin in the AE1 multimolecular membrane complexes at the erythrocyte membrane. Using mass spectrometry of the isolated cross-linked stomatin complexes from erythrocytes, Rungaldier *et al*.^10^ proposed that AE1 could be an interaction partner of stomatin in RBCs. More recently, by proteomic analysis, stomatin was found to be present in the erythrocyte membrane skeleton including AE1 complexes^38^. The relationship between stomatin and AE1 was demonstrated here by Proximity Ligation Assay (PLA) on a previously described HEK293 cell system expressing recombinant eAE1 after tetracycline induction^39^. Moreover, an adaptation of the PLA for the first time to RBCs allowed the detection *in situ* of an interaction between stomatin and the erythroid chloride/bicarbonate exchanger (eAE1). Regarding a decrease of PLA efficiency in RBCs compared to other cells, in particular recombinant HEK293 studied here, one explanation may lie in the presence of hemoglobin which is known to inhibit reactions of DNA polymerization^40^. Nevertheless, PLA on RBCs, developed in the present study, enables the revelation of *in situ* interactions or at least proximity (<40 nm) between membrane proteins and cytoskeleton partners or other membrane proteins. This new approach on RBCs provides a qualitative method of describing the interactions within RBCs membrane complexes.

Which among the different forms of AE1 (free dimers or anchored to the spectrin-based skeleton within the ankyrin complex, as tetramers, or within the junctional complex, as dimers) are involved in AE1-stomatin complexes cannot be easily identified. Previous work on the expression of recombinant AE1 in HEK293 cells^41^ identified the presence of oligomeric forms of AE1 at the plasma membrane, but never more complex than dimers. In this study, PLA results in the recombinant AE1 expressing cells indicate dimers of AE1 as possible partners of stomatin. Moreover, while recombinant kAE1 was recently shown to be able to bind to endogenous ankyrin G which corresponds to the only form of ankyrin found in HEK293 cells, recombinant eAE1 expressed in the same cells cannot bind to the endogenous ankyrin G^39^. This suggests that tetrameric forms of eAE1 are absent from the membrane of these recombinant cells. Since ankyrin R expression is restricted to erythrocytes, we were able to detect, as expected from the well-known eAE1-ankyrin R interaction^17^, a positive PLA signal on RBCs when anti-AE1 and anti-ankyrin R antibodies were used. However, PLA was found negative when an interaction between ankyrin R and stomatin was tested (not shown), suggesting that stomatin is not present in the ankyrin complex or is at least too distant from ankyrin R (>40 nm). In contrast, a stomatin-actin association was previously reported by a study which was focused on the composition of membrane rafts and vesicles in stomatin-deficient OHSt RBCs^42^, suggesting that stomatin is present in the junctional complex. The authors also showed that a recombinant expression of stomatin in MDCK increases the actin association to the plasma membrane. Taking into account these data together with the well-kwown eAE1-actin interaction^17^, the stomatin-actin association is consistent with the binding of stomatin to the dimeric forms of AE1 within the junctional complex and not to the tetrameric form in the ankyrin complex.

**Figure 8:**
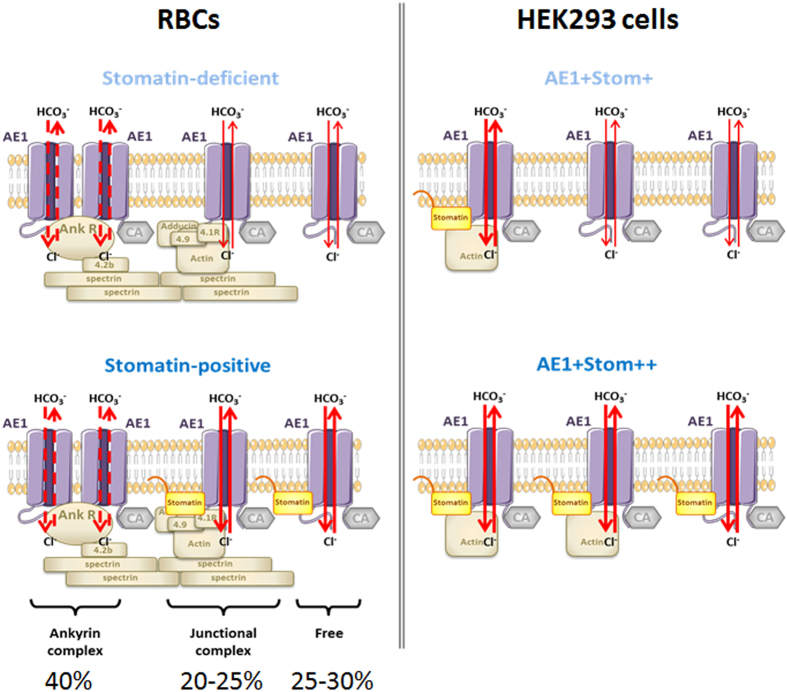
Putative model of stomatin interaction with AE1 in RBCs and HEK293 cells and consequences on the activity of AE1. In RBCs (left panel), in the absence of stomatin (stomatin-deficient), the chloride bicarbonate exchange (red arrows) is reduced in dimeric forms of AE1 (free or engaged in the junctional complex). In HEK293 cells expressing AE1 (right panel), the overexpression of stomatin is associated with an increase of AE1 activity.

Among stomatin partners, GLUT1 was the first described with modifications of its activity depending on its interaction with stomatin^11^. Although an interaction between AQP1 and stomatin was suggested by mass spectrometry of isolated cross-linked stomatin complexes^10^ and confirmed here by PLA (Fig. 2S), water transport was found to be unchanged in absence of stomatin^12^. In the present study, the function of AE1 could be investigated in the stomatin-deficient RBCs using stopped-flow approaches, showing a two-fold slower chloride/bicarbonate exchange activity across the membrane while the expression level of AE1 at the RBC plasma membrane was unmodified in these patients compared to controls. This decrease was demonstrated in resealed ghosts not only by intracellular pH (pHi) measurement after bicarbonate influx, as we reported in a previous study of AE1 in a HSt patient^31^, but also by chloride efflux, following a new approach adapted from Illsley and Verkman^35^. The around 50% alteration of AE1 activity in stomatin-deficient RBCs suggests that the different forms of AE1 might not be equally linked and sensitive (in terms of transport activity) to stomatin. Suggested by the negative PLA result for interaction between ankyrin R and stomatin and in agreement with the previously described actin-stomatin association in RBCs^42^, we propose that dimeric forms of AE1 (free or engaged in the junctional complex) rather than tetrameric ones (within the ankyrin complex) could be those which are impaired in stomatin-deficient RBCs (Fig. 8)

The activity of recombinant AE1 was also tested in HEK293 cells overexpressing recombinant stomatin. In these cells which express endogenous stomatin and carbonic anhydrase, kinetics of pHi changes could be assessed by stopped-flow by submitting the cells to a bicarbonate gradient, as previously described^31^. Interestingly, the overexpression of stomatin led to an increase of AE1 activity while the surface expression level of the exchanger was unchanged when compared to cells which only express endogenous stomatin. This suggests that the expression level of endogenous stomatin is not sufficient or adequate to optimize the activity of large amounts of recombinant AE1 at the plasma membrane of HEK293 cells. Furthermore, PLA testing the AE1-stomatin interaction in HEK293 cells, with equivalent AE1 surface expression after tetracycline induction, revealed a significant increase of the number of spots when cells overexpressed stomatin. This suggests that some free dimers of recombinant AE1 not yet engaged into the junctional complex but expressed at the membrane of transfected and induced HEK293 cells could be recruited by recombinant stomatin, most likely in association with actin. Such an actin-stomatin-AE1-complex in recombinant cells might be similar to that expressed at the RBCs membrane, supporting the impact of stomatin on dimeric forms of AE1. The recruitment of AE1 by stomatin has a direct impact on the activity of the exchanger in the recombinant cells (Fig. 8).

In conclusion, from the analysis of both stomatin-deficient RBCs and recombinant cells overexpressing stomatin, the present study gives an evidence of not only a physical link between AE1 and stomatin, as previously suggested^10^, but also a functional relationship between the exchanger and a new partner of the AE1 membrane complex currently considered as an integral scaffolding protein of lipid rafts^43^. Further studies in normal RBCs placed under stress conditions will be necessary to evaluate to which extent modifications of the physical interactions between AE1 and stomatin result in a reduction of AE1 activity with consequences on intracellular pH and volume homeostasis.

## Methods

### Reagents

Rabbit polyclonal antibody anti-AE1 was kindly provided by Dr C. Wagner (University of Zürich, Switzerland). Mouse monoclonal antibody anti-AE1 (BRIC6) came from the International Blood Group Reference Laboratory (IBGRL, Bristol, United Kingdom). Mouse monoclonal antibodies anti-ankyrin R (clone N388A/10) and anti-AQP1 (clone 1/A5F6) were from Neuromab (UC Davis/NIH, USA) and Bio-Rad (Marnes-la-Coquette, France) respectively, while goat polyclonal anti-stomatin (M-14) and rabbit polyclonal anti-CAII antibodies were purchased from Santa Cruz Biotechnology (Dallas, Texas USA). Secondary antibodies used in flow cytometry analysis were phycoerythrin (PE) or fluorescein isothiocyanate (FITC)-conjugated F(ab’)_2_ fragment of goat anti-mouse and donkey anti-goat immunoglobulins from Beckman Coulter (Villepinte, France). BCECF-AM [2′,7′bis-(2-carboxyethyl)-5(6)-carboxyfluorescein acetoxymethyl ester] and pyranine [8-hydroxypyrene-1,3,6-trisulfonic acid] fluorescent probes, as well as carbonic anhydrase from bovine erythrocytes and the AE1 inhibitor DIDS [4,4 2-Diisothiocyanatostilbene-2,2 2-disulfonic acid disodium salt] were purchased from Sigma-Aldrich (Saint Quentin, France). Chloride indicator SPQ (6-methoxy-N-(3-sulfopropy1) Quinolinium) was obtained from Invitrogen (Fisher Scientific, Illkirch, France).

### Blood samples

RBCs used for ghost preparations correspond to blood samples described in a previous study^12^ and originally collected after informed consent obtained from all participants and kept frozen at the Centre National de Référence des Groupes Sanguins (CNRGS). This study was approved and conducted according to institutional ethical guidelines of the National Institute for Blood Transfusion (INTS, Paris, France).Before analysis, cryopreserved RBCs were washed three times with phosphate-buffered saline (PBS).

### Construction of the stomatin expression vector

Stomatin was amplified by PCR from human bone marrow cDNAs (Takara Bio Europe/Clontech, Saint Germain-en-Laye, France) using the following primers; sens 5′-GGGAATTCAAGCTTGCCGCCACCATGGCCGAGAAGCGGCA-3′ and antisens 5′-GGGAATTCGCGGCCGCCTAGC CTAGATGGCTGTG-3′. Primers contain restriction sites *Hind* III and *Not* I, respectively (underlined sequences). Using Phusion HF DNA polymerase (Thermo Fisher Scientific, Villebon sur Yvette, France), PCR amplification assay was performed as followed: 98 °C 30 sec, 98 °C 10 sec, 58 °C 10 sec, 72 °C 30 sec (30 cycles), 72 °C 30 sec. PCR product was then cloned into pCEP4 expression vector. The final construction was proof-sequenced (GATC Biotech, Konstanz, Germany) before starting experiment.

### Cell culture, transfection with pCEP4-stomatin and flow cytometry analysis

The tetracycline-inducible AE1 transfected cell line HEK293-AE1^31^ was maintained at 37 °C in DMEM-F12-Glutamax (Invitrogen) supplemented with 1% NEAA (Invitrogen), 10% tetracycline-depleted fetal calf serum (Gibco), 25 mM Hepes (Invitrogen), 25 mM NaHCO_3_, 5 μg/ml blasticidin and 25 μg/ml zeocin. AE1 expression was induced by addition of 1 μg/ml tetracyclin 16 h before experiments. The HEK293-AE1 cell line was transfected with plasmid pCEP4-stomatin in presence of FuGENE 6 (Roche Diagnostics GmbH, Mannheim, Germany) and Opti-MEM (Invitrogen). Stable expression of recombinant stomatin was obtained by addition of 300 μg/ml hygromycin (Invitrogen) to the culture medium. Expression of AE1 at the surface of non-permeabilized HEK293 cells and of stomatin after cell permeabilisation was detected with a FACSCanto II (BD Biosciences, San Jose, CA, USA) after staining with the relevant primary antibodies. Cells were gated on dot plots (SSC-A vs FSC-A), as shown in Fig. 3S. Four types of cells were thus generated: endogenous stomatin-expressing cells, with or without tetracycline-induction of AE1 (AE1+ Stom+ or AE1-Stom+), and recombinant stomatin-expressing cells, with or without tetracycline-induction of AE1 (AE1+ Stom++ or AE1-Stom++).

### Measurement of AE1 activity by two stopped-flow assays

The first assay which is based on intracellular pH changes was performed on recombinant HEK293 cells expressing AE1 and on ghosts derived from RBCs, as previously described^31^. Briefly, recombinant cells and “pink ghosts” were first incubated or resealed in the presence of KCl with a pH-sensitive probe (BCECF-AM for cells and pyranine for ghosts) and carbonic anhydrase (only for ghosts). They were then submitted to a KHCO_3_ gradient, thus creating entering HCO_3_^−^ and exiting Cl^−^ gradients. The pH-dependent fluorescence changes of BCECF and pyranine were monitored at a 485 nm and a 465 nm excitation wavelength (30 °C), respectively, and the emitted light was filtered with a 520 nm cut-off filter. The second assay, which is based on chloride concentration changes and carried out on ghosts derived from RBCs, was adapted from a method previously described by Illsley and Verkman^35^. In the present study, ghosts were resealed in 50 mM KCl, 50 mM KGluconate, 100 mM sucrose, 50 mM Hepes/Tris pH 7.2 in the presence of 10 mM SPQ, a fluorescent probe that detects Cl^−^ via diffusion-limited collisional quenching. The resealed ghosts were then mixed vol/vol with the bicarbonate buffer (100 mM KHCO_3_, 100 mM sucrose, 50 mM Hepes/Tris pH 7.2) in the reaction chamber at 20 °C, thus again creating entering HCO_3_^−^ and exiting Cl^−^ gradients. The kinetics of fluorescence increase, corresponding to the probe dequenching by decrease of the chloride concentration in the ghosts, were monitored at a 365 nm excitation wavelength and the emitted light was filtered with a 450 nm cut-off filter. Data from three to eight time-courses corresponding to kinetics of fluorescence increases were averaged and the experimental curves fitted to a mono-exponential function to estimate either the alkalinisation or the probe dequenching rate constants k (s^−1^), using the simplex procedure of the Biokine software package (Bio-Logic, Claix, France). The mono-exponential equations are : (HCO_3_^−^)_t_^int^ = (HCO_3_^−^) ^ext^. (1-exp^(−kt)^) and (Cl^−^)_t_^ext^ = (Cl^−^) ^int.^(1-exp^(−kt)^), where k is the rate constant of the exponentials.

### Ghost radius measurement

Ghosts prepared as described above were incubated in the appropriate chloride buffer supplemented with 0.5 mg/ml eosin-5-maleimid (Life Technologies, Fisher Scientific, Illkirch, France) for 1 h. For each sample, 300 ghosts were analysed under a LSM 710 microscope (Zeiss), with a fluorescence excitation wavelength at 450 nm. Mean radius value thus determined was used to calculate volume (V), surface area (SA) and apparent permeability P’ following the formula P’ _HCO3_^−^ = k x V/SA, where k is the alkalinisation rate constant obtained from stopped-flow experiments.

### Proximity ligation assay

Proximity ligation assay (PLA) was used for *in situ* detection of protein interactions. HEK293-AE1 cells, expressing or not recombinant stomatin, were cultured on polylysine coverslips, induced or not for AE1 expression as described above. Cells on coverslips were fixed, permeabilized, incubated with antibodies raised in different species and then submitted to different mixtures for ligation and amplification according to the manufacturer’s instructions (Olink Bioscience, Uppsala, Sweden), as previously described. Antibodies used were rabbit anti-AE1 (1:4000) and goat anti-stomatin (1:50) antibodies. In the present study, we adapted the PLA to RBCs. RBCs were washed in PBS, fixed with 1% formaldehyde/0.025% glutaraldehyde, permeabilized in 1% octylglucopyranoside, then projected on polylysine coverslips by cytospin centrifugation (5 min at 800 rpm). Coverslips were then blocked in background reducing reagent (Dako, Denmark) and treated as described above. Antibodies used here were rabbit anti-AE1 (1:4000) and goat anti-stomatin (1:25) or mouse anti-ankyrin R (1:2), anti-AQP1 (1:100) and anti CAII (1:60) antibodies. Cells and RBCs were analysed by confocal microscopy. Red dots indicating an interaction are produced when the two tested protein are <40 nm apart. They were quantified by ImageJ software.

## Additional Information

**How to cite this article:** Genetet, S. *et al*. Stomatin modulates the activity of the Anion Exchanger 1 (AE1, SLC4A1). *Sci. Rep.* **7**, 46170; doi: 10.1038/srep46170 (2017).

**Publisher's note:** Springer Nature remains neutral with regard to jurisdictional claims in published maps and institutional affiliations.

## Supplementary Material

Supplementary Information
