# Supplementary material for: Stomatin modulates the activity of the Anion Exchanger 1 (AE1, SLC4A1)
Source: Sci Rep. 2017 Apr 7;7:46170. doi: 10.1038/srep46170 (PMC5383999; doi:10.1038/srep46170)
Supplement: Supplementary Information [file srep46170-s1.pdf]

Stomatin modulates the activity of the Anion Exchanger 1 (AE1, SLC4A1)

Sandrine Genetet, Alexandra Desrames, Youcef Chouali, Pierre Ripoche, Claude Lopez and Isabelle Mouro-Chanteloup

Supplementary information

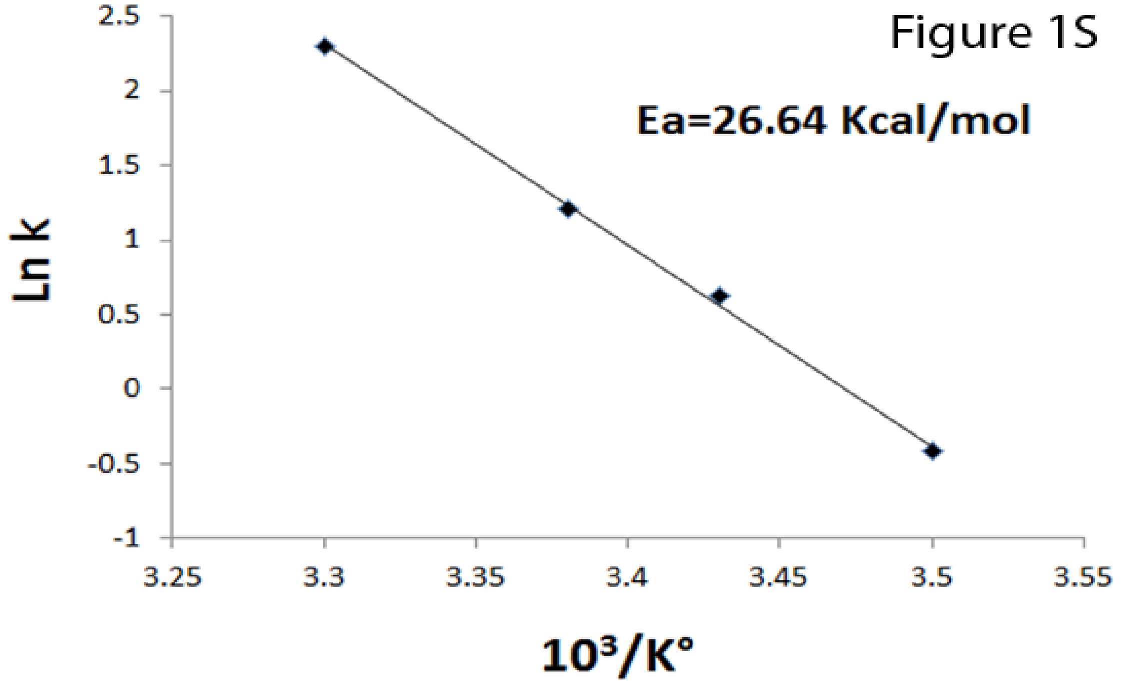

Figure 1S: Arrhenius activation energy (Ea) for the chloride permeation in ghosts derived from control RBCs. Ea was deduced from the Arrhenius plot of temperature-dependent chloride efflux. The coefficient of determination R^2 is 0.9984.

Figure 2S

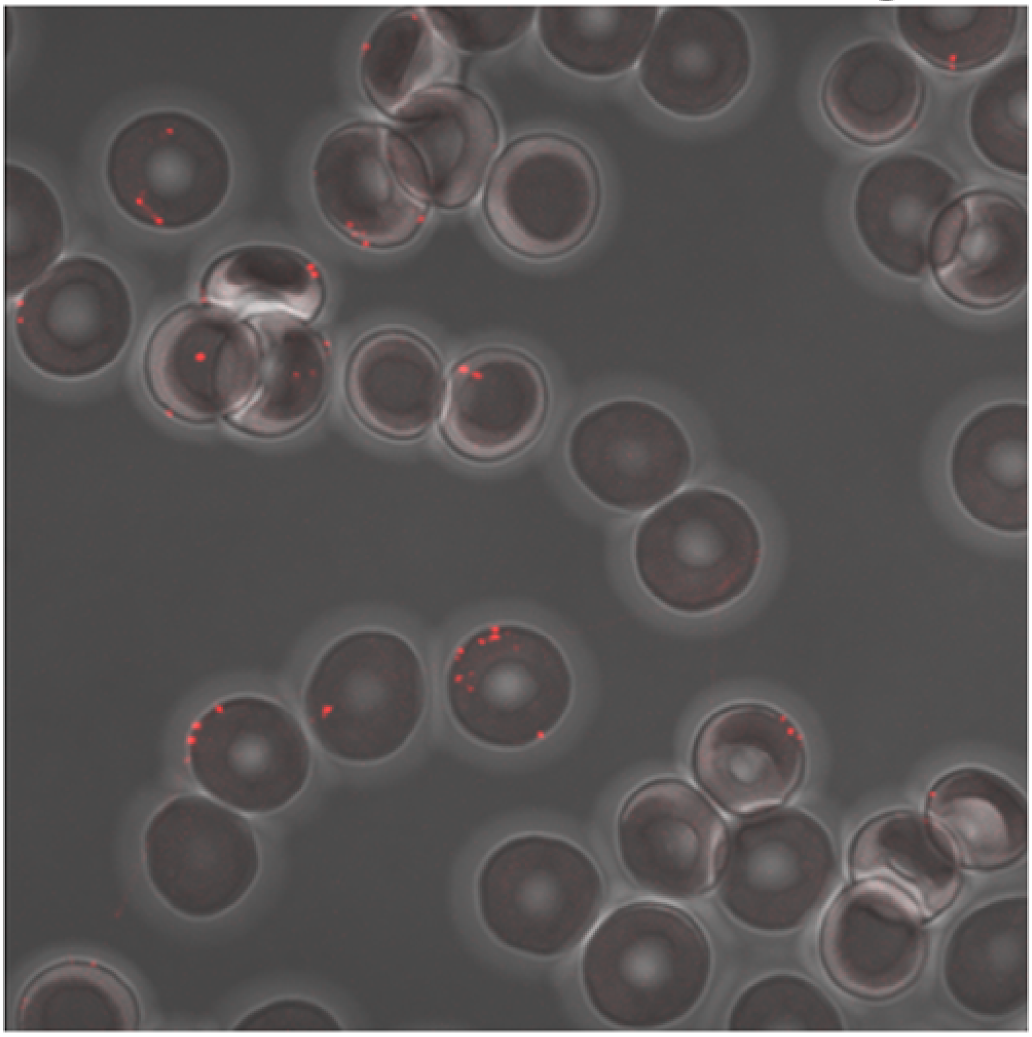

Figure 2S: Proximity ligation assays for protein interaction between AQP1 and stomatin. RBCs from a control (stomatin-positive) were treated as described in experimental procedures, using anti-AQP1 along with anti-stomatin antibodies. RBCs were then examined by confocal microscopy using a Zeiss LSM700 inverted confocal microscope equipped with a x100 oil-immersion objective, numerical aperture 1.4. Z-stack confocal image capture was performed and analyzed using the ZEN software.

Figure 3S

A

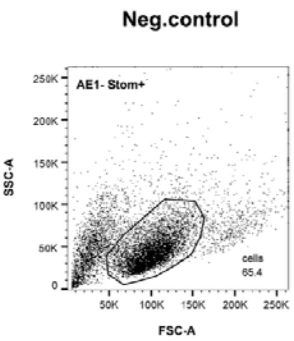

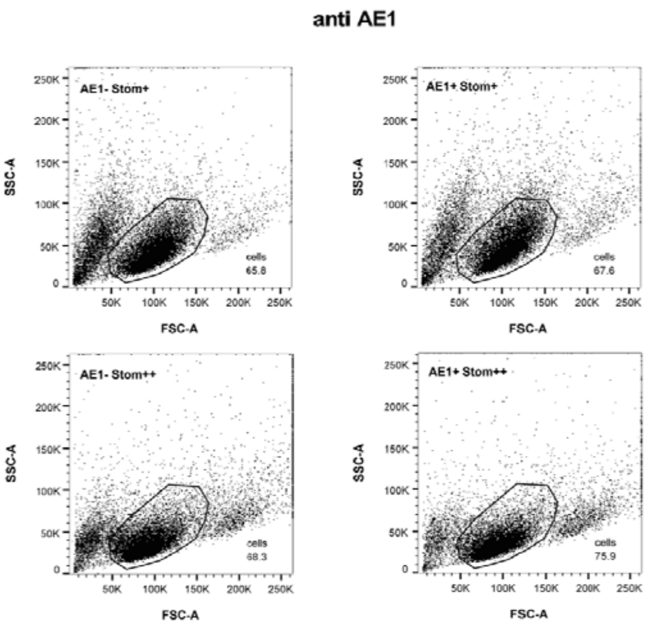

cells	Freq of Parent	Geo Mean (FITC-A)
Neg. Control	74.0 %	36.6
AE1- Stom+	74.2 %	514
AE1+ Stom+	81.1 %	409
AE1- Stom++	72.5 %	871
AE1+ Stom++	79.1 %	842

B

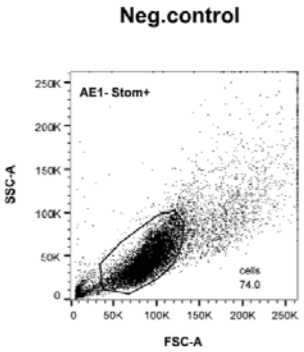

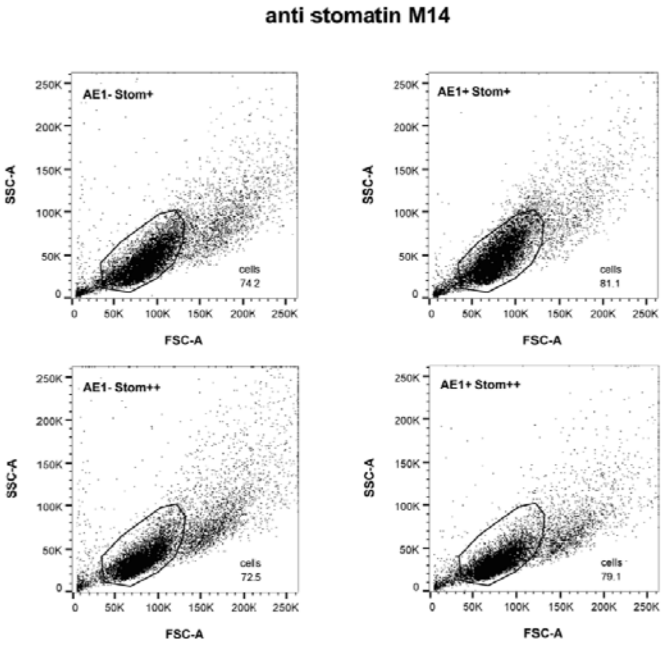

cells	Freq of Parent	Geo Mean (FITC-A)
Neg. Control	65.4 %	87.6
AE1- Stom+	65.8 %	265
AE1+ Stom+	67.6 %	7317
AE1- Stom++	68.3 %	257
AE1+ Stom++	75.9 %	7258

Figure 3S: SSC-A vs FSC-A dot plots. From a gated population of cells representing about 70% (Freq of parent) of total cells, 10.000 events were analysed. Geometric Means were determined on these cells. (A) AE1 expression using anti-AE1 antibody. (B) Stomatin expression using M-14 antibody.
